# The therapeutic effect of traditional Chinese medicine on breast cancer through modulation of the Wnt/β-catenin signaling pathway

**DOI:** 10.3389/fphar.2024.1401979

**Published:** 2024-05-09

**Authors:** Hongkun Li, Jiawei Li, Yifan Zhang, Chengcheng Zhao, Jun Ge, Yujiao Sun, Hui Fu, Yingpeng Li

**Affiliations:** ^1^ College of Chinese Materia Medica, Tianjin University of Traditional Chinese Medicine, Tianjin, China; ^2^ College of Acupuncture-Moxibustion and Tuina, Tianjin University of Traditional Chinese Medicine, Tianjin, China; ^3^ Experimental Teaching and Practical Training Center, Heilongjiang University of Chinese Medicine, Harbin, China; ^4^ College of Integrated Chinese and Western Medicine, Tianjin University of Traditional Chinese Medicine, Tianjin, China

**Keywords:** breast cancer, Wnt/β-catenin signaling pathway, traditional Chinese medicine, chemotherapy drugs, therapeutic approach

## Abstract

Breast cancer, the most prevalent malignant tumor among women globally, is significantly influenced by the Wnt/β-catenin signaling pathway, which plays a crucial role in its initiation and progression. While conventional chemotherapy, the standard clinical treatment, suffers from significant drawbacks like severe side effects, high toxicity, and limited prognostic efficacy, Traditional Chinese Medicine (TCM) provides a promising alternative. TCM employs a multi-targeted therapeutic approach, which results in fewer side effects and offers a high potential for effective treatment. This paper presents a detailed analysis of the therapeutic impacts of TCM on various subtypes of breast cancer, focusing on its interaction with the Wnt/β-catenin signaling pathway. Additionally, it explores the effectiveness of both monomeric and compound forms of TCM in the management of breast cancer. We also discuss the potential of establishing biomarkers for breast cancer treatment based on key proteins within the Wnt/β-catenin signaling pathway. Our aim is to offer new insights into the prevention and treatment of breast cancer and to contribute to the standardization of TCM.

## Highlights

• Elucidated the complex role of TCM in treating breast cancer through the Wnt/β-catenin signaling pathway, emphasizing its dual capacity to modulate molecular mechanisms and enhance therapeutic potential when integrated with conventional therapies.
• Advanced a framework for TCM standardization focused on rigorous quality control, the use of biomarkers for clinical efficacy validation, and the synergy of combining TCM with established therapeutic modalities to reduce adverse effects.

## 1 Introduction

Breast cancer, stemming from the epithelial tissue of the mammary gland, has witnessed a steady surge in its incidence in recent years (Ni et al., 2022; Guo et al., 2023; Patel et al., 2023), solidifying its position as the foremost cancer afflicting women (Hashemi et al., 2023). As we delve deeper into the intricacies of this disease, it becomes evident that the Wnt signaling pathway stands at the crossroads of our understanding. Over the past decades, extensive research has illuminated the central role this pathway plays in breast cancer, influencing processes such as proliferation (Wend et al., 2013), metastasis (Luga et al., 2012; Harper et al., 2016), stem cell maintenance (Wang et al., 2016), drug resistance (Piva et al., 2014), and phenotyping (Piva et al., 2014; Jiang et al., 2019). It has been found that the Wnt/β-catenin axis is the core component of the Wnt signaling pathway, making the design of targeted therapy for it a growing research focus (Hernandez et al., 2012; Nishiya, 2017). However, despite the evident anti-cancer potential of inhibitors targeting this axis (Kahn, 2014), their translation from research labs to clinical application has encountered numerous obstacles. Challenges such as off-target effects (Ayadi et al., 2015), potential toxicity (Zhong et al., 2016; Wan et al., 2021), and the intricate nature of the Wnt/β-catenin signaling (Zimmerli et al., 2017) have been formidable barriers. This complexity is further underscored by the fact that no inhibitors specifically targeting the Wnt/β-catenin axis have been approved for breast cancer treatment (Zimmerli et al., 2017; Xu et al., 2020). As a result, conventional methods like surgical intervention, radiotherapy, and chemotherapy remain the primary recourse, despite their often suboptimal outcomes (Li et al., 2023).

**FIGURE 1:**
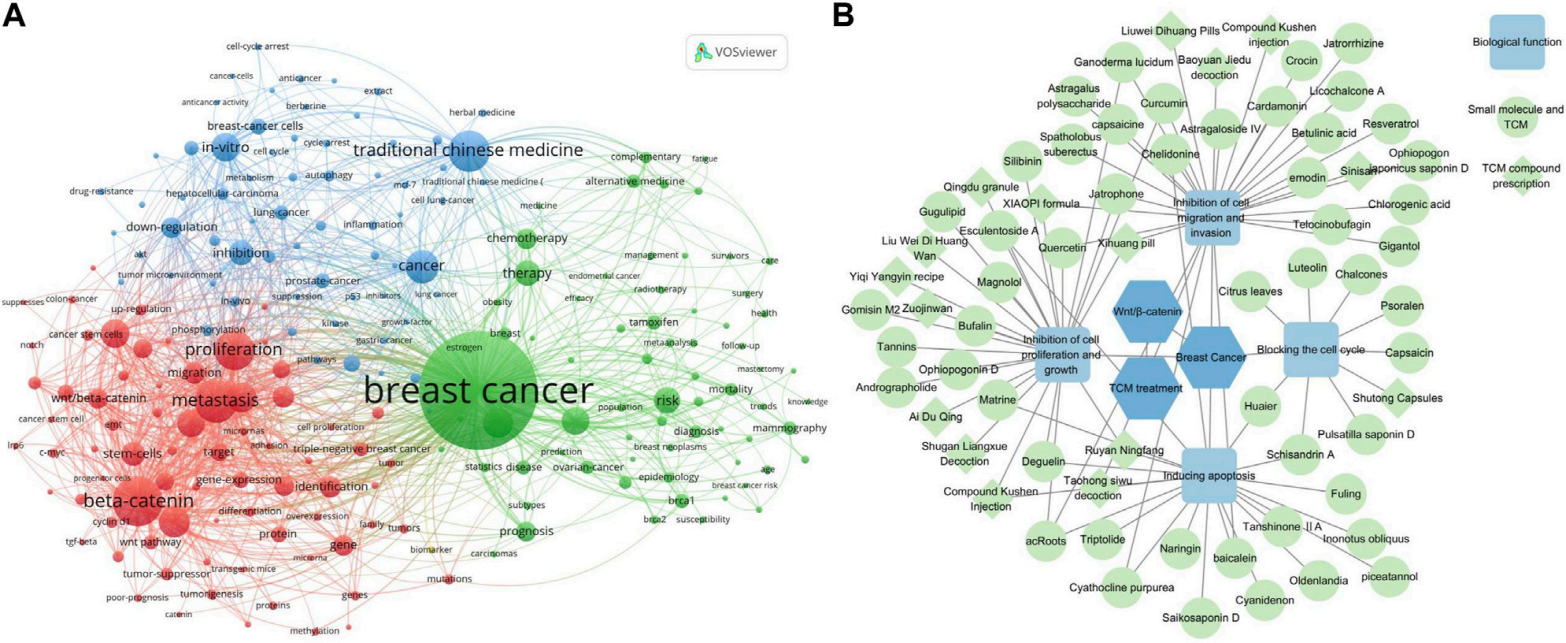
**(A)** Analysis using VOSviewer of key terms in breast cancer research, highlighting the prominence of TCM and the Wnt/β-catenin signaling pathway as current focal areas of investigation. **(B)** Network diagram crafted with Cytoscape software, illustrating the intricate interplay between various TCMs and their roles in breast cancer treatment. In this visualization, individual small molecule and TCM are denoted by circles, compound medicines by diamonds, and their corresponding biological functions by light blue squares. This representation not only elucidates the potential mechanisms of action of TCM, such as cell cycle blockade, inhibition of cell proliferation and growth, suppression of cell migration and invasion, and induction of apoptosis but also underscores the profound connection between TCM, breast cancer, and the pivotal Wnt/β-catenin signaling pathway.

Amidst this backdrop, TCM emerges as a beacon of hope. Its therapeutic potential is intricately linked with the modulation of the Wnt/β-catenin signaling pathway. Clinical studies have consistently emphasized the significant role TCM plays in breast cancer management, attributed to its low toxicity and comprehensive protective effects (Guo et al., 2020; Wang S. Y. et al., 2023). The diverse active metabolites within TCM not only interact with various cellular pathways but are especially adept at modulating the Wnt/β-catenin axis (Alharbi et al., 2022). This synergy between TCM, breast cancer, and the Wnt/β-catenin pathway has ignited a surge in research endeavors, examining TCM’s efficacy both as an independent therapeutic approach and in tandem with chemotherapy agents, as depicted in Figure 1A.

This paper endeavors to provide a comprehensive overview of the unique attributes of the Wnt/β-catenin signaling pathway in breast cancer. Additionally, we delve into the potential of Chinese medicine monomers and compounds as therapeutic agents, specifically targeting the Wnt/β-catenin signaling pathway components (Figure 1B). The insights garnered from this study serve as a pivotal reference for future breast cancer research, paving the way for the evolution of novel therapeutic strategies tailored to different disease subtypes.

## 2 Traditional understanding and treatment of breast cancer in TCM

In clinical practice for early-stage breast cancer (stages I and II), treatments often involve modified radical mastectomy or breast-conserving surgery. However, these surgical procedures can cause considerable physical trauma and potentially impair limb function in patients. For more advanced stages (III and IV), chemotherapy is the typical course of action. This treatment regimen includes a combination of various drugs, such as anthracyclines, antimetabolites, alkylating agents, and platinum compounds (Marsh and Liu, 2009). However, most of these chemotherapy drugs are associated with severe side effects, including gastrointestinal disturbances, bone marrow suppression, ovarian and thyroid toxicity, and the development of drug resistance (Mortezaee et al., 2019; Zhou et al., 2022). In contrast, TCM adopts a holistic approach, focusing on syndrome differentiation and tailoring special prescriptions for specific diseases. Traditional chinese medicine aims to regulate the balance of yin and yang, harmonize qi (vital energy), and foster positive health trends. It has shown potential in treating breast cancer and its complications, mitigating adverse reactions to chemotherapy, enhancing postoperative recovery, and reducing the risk of recurrence and metastasis. Given its integration with modern science and technology and its synergistic use with Western medicine, TCM is increasingly recognized as a vital component in the comprehensive treatment of breast cancer.

Although there is no specific term for “breast cancer” in TCM, ancient Chinese medical literature often refers to it as “Ruyan” or “Mammary Mastitis.” It is traditionally linked to internal pathogenic factors such as qi stagnation, blood stasis, and phlegm turbidity. As articulated in the “Required reading for medical practitioners (医宗必读),” it is mentioned that “when the healthy qi is deficient, the pathogenic qi accumulates (正气不足而后邪气聚之)” and “where pathogenic factors converge, qi is invariably weak (邪之所凑, 其气必虚)” as cited in “Su Wen Comment on Fever (素问·评热病论篇).” This underscores the Chinese medicinal philosophy that while external factors might instigate the onset of breast cancer, it is the internal causes that play a pivotal role. Both these factors, in tandem, lead to the disease’s manifestation. Consequently, TCM’s approach to breast cancer treatment is holistic, emphasizing both local and systemic treatments, underpinned by dialectical reasoning. The primary therapeutic strategies encompass bolstering body resistance, purging pathogenic factors, detoxifying to eliminate carbuncles, and promoting blood circulation to remove stasis.

Several TCM compounds, such as *ShuTong* Capsule (Du et al., 2014), *XiaoPi* formula (Zheng et al., 2020), *BaoYuanJieDu* Tang (Tian et al., 2020), *QingDu* granule (Zhao et al., 2018), and Compound *KuShen* Injection (Nourmohammadi et al., 2019), which have their roots in ancient Chinese medical scriptures, have shown promising clinical results in breast cancer treatment. Concurrently, the main TCM in these prescriptions, including Ganoderma lucidum (Leyss.ex Fr.) Karst (*Ganoderma lucidum*) (Zhang, 2017), Astragalus mongholicus Bunge (Fabaceae; Astragali radix) (Jiang et al., 2017), Andrographis paniculata (Burm.f.) Wall. ex Nees (Acanthaceae; Andrographis herba) (Xu et al., 2022), Scleromitrion diffusum (Willd.) R. J. Wang (Rubiaceae; Hedyotis diffusa willd) (Li et al., 2017), *Bupleurum chinense* DC. (Apiaceae; Bupleuri radix) (Wang J. X. et al., 2018), *Salvia miltiorrhiza* Bunge (Lamiaceae; Salviae miltiorrhizae radix et rhizoma) (Zhao H. et al., 2022), *Angelica sinensis* (Oliv.) Diels (Apiaceae; Angelicae sinensis radix) (Zhu et al., 2021), Sophora flavescens Aiton (Fabaceae; *Sophorae fiavescentis* radix) (Li et al., 2020), and *Glycyrrhiza uralensis* Fisch. ex DC. (Fabaceae; Glycyrrhizae radix et rhizoma) (Bortolotto et al., 2017), have been the subject of rigorous research. These TCM have consistently demonstrated potent anti-breast cancer properties. Further studies have pinpointed that the therapeutic efficacy of these individual Chinese medicine components against breast cancer is largely attributed to their active metabolites, such as Astragalus polysaccharide (Yang S. et al., 2020), Astragaloside IV (Jiang et al., 2017), Saikosaponin D (Wang J. X. et al., 2018), Tanshinone ⅡA (Li and Lai, 2017), Matrine (Xiao et al., 2018), Capsaicin (Wu D. et al., 2020), Betulinic acid (Zheng Y. F. et al., 2019) and more.

Through numerous *in vitro* and *in vivo* experiments, it has been discovered that Chinese medicine compounds, monomers, and metabolites can inhibit the growth, migration, invasion, apoptosis, and recurrence of breast cancer by regulating Wnt/β-catenin, NF-κB, MAPK, and PI3K/AKT pathways. Through the study of network pharmacology, we found that these signaling pathways are closely related to the Wnt/β-catenin pathway. In breast cancer cells, NF-κB activation can inhibit β-catenin/TCF activity, thus negatively regulating the Wnt/β-catenin pathway (Ma and Hottiger, 2016). At the same time, the key activators of the NF-κB pathway, IKKα and IKKβ, can interact with β-catenin and phosphorylate it to positively regulate the Wnt/β-catenin pathway (Lamberti et al., 2001). Key members of the MAPK signaling pathway, such as ERK1/2, p38MAPK, and JNK, are involved in the phosphorylation of low-density lipoprotein receptor-related protein 6 (LRP6), which stimulates the expression of β-catenin, thereby influencing the Wnt/β-catenin signaling pathway (Zhang et al., 2014). There are many common connecting elements between the PI3K/AKT and Wnt/β-catenin pathways, such as GSK3β, FZD, DVL, Deptor, and eIF4E, which can interfere with the key process of regulating β-catenin degradation and β-catenin nuclear translocation (Gingras et al., 1999; Prossomariti et al., 2020). Based on these studies, we found that the mechanism of TCM in treating breast cancer is related to Wnt/β-catenin. Therefore, Wnt/β-catenin plays a crucial role in the interaction between TCM and breast cancer.

## 3 Wnt/β-catenin signaling and its interplay with TCM

**FIGURE 2:**
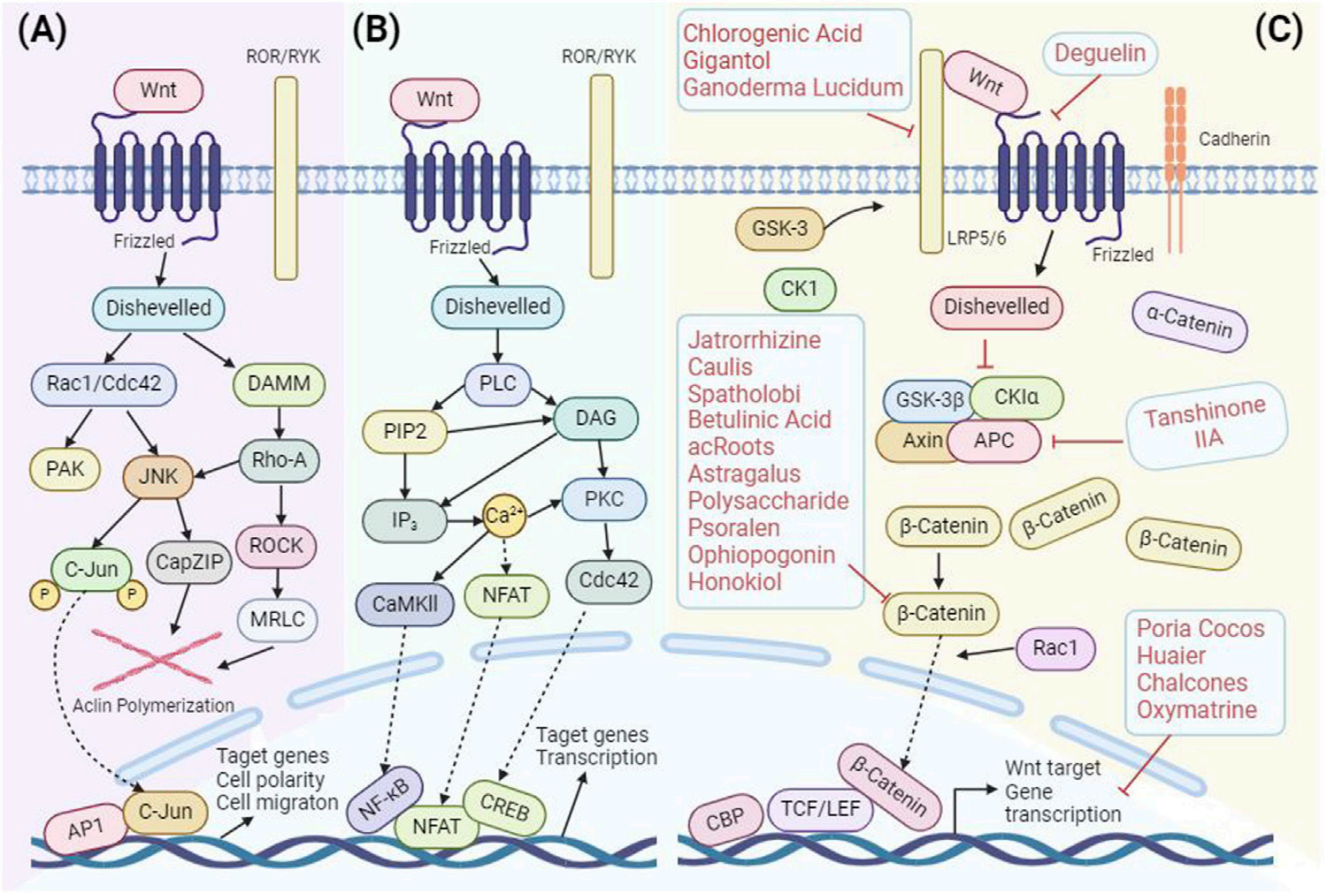
Diagrammatic representation of Wnt Signaling Pathways in Mammals: **(A)** Wnt–PCP pathway, **(B)** Wnt–Ca^2+^ pathway, and **(C)** Canonical Wnt pathway, with highlighted target positions modulated by TCM.

The Wnt protein family, a collection of secreted lipid-modified glycoproteins (Oh et al., 2023), is instrumental in orchestrating a range of biological activities, from cell adhesion (Schambony et al., 2004; Astudillo and Larrain, 2014) and migration (Tuttle et al., 2014; Chai et al., 2019) to proliferation (Tang et al., 2019; Bertozzi et al., 2022), differentiation (Matsumoto et al., 2023), and survival (Jia et al., 2019). The Wnt signaling cascade bifurcates into two main branches: the canonical or classical Wnt pathway (Figure 2), commonly referred to as the Wnt/β-catenin pathway, and the non-canonical Wnt pathway, which further branches into the Wnt/planar cell polarity (PCP) pathway and the Wnt/Ca^2+^ pathway (Koni et al., 2020; Azbazdar et al., 2021; Patel et al., 2023) (Figures 2A, B). While these pathways have varying dependencies on β-catenin, together they form an intricate regulatory network.

The Wnt/β-catenin signaling pathway, omnipresent in cells, is pivotal in modulating inflammatory reactions (Yang et al., 2022), lessening fibrosis (Mo et al., 2015), and counteracting osteoporosis (Zhang et al., 2017). Its significance is further underscored by its role in the etiology of various diseases. Traditional Chinese Medicine has shown potential in modulating this pathway. For example, *HuangLianJieDu* Decoction has been observed to attenuate the expression of LRP5/6, Wnt1, and β-catenin, thereby intervening at different junctures of the Wnt/β-catenin signaling pathway. This modulation has proven beneficial in treating conditions like psoriasis (Yang et al., 2022). Similarly, the *HuangGan* recipe acts by dampening the expression of Wnt1, β-catenin, and transcription factor 4, offering therapeutic advantages in conditions like glomerulosclerosis and tubulointerstitial fibrosis (Mo et al., 2015). Another notable mention is the *BuShenJianPiHuoXue* Decoction, which, in the context of diabetic osteoporosis, activates the Wnt/β-catenin signaling pathway and concurrently inhibits the NF-кB signaling pathway (Zhang et al., 2017). In essence, the intricate dance between TCM and the Wnt/β-catenin signaling pathway offers promising avenues for therapeutic interventions in a myriad of diseases, emphasizing the profound connection between TCM and the Wnt/β-catenin pathway.

Building on the intricate relationship between TCM and the Wnt/β-catenin signaling pathway, it is imperative to delve deeper into the pathway’s role in breast cancer. The Wnt/β-catenin pathway is intrinsically linked to the onset and progression of breast cancer.

Compared with traditional chemotherapy and radiotherapy, small molecular inhibitors are considered to be the most promising therapeutic strategies targeting the Wnt/β-catenin signaling pathway. These inhibitors are mainly divided into Wnt ligands inhibitors [e.g., WNT974 (Solzak et al., 2017), Wnt-C59 (Proffitt et al., 2013), and RXC004 (Shah et al., 2021)], Wnt receptor inhibitors [e.g., Mesd (Ma et al., 2017), Niclosamide (Lu et al., 2011), and Dickkopf1 (Bu et al., 2008)] β-catenin destruction complex inhibitors [e.g., E7449 (McGonigle et al., 2015; Asano et al., 2018; Plummer et al., 2020), XAV939 (Fang et al., 2021), and Pyrvinium (Rodgers et al., 2023)] and anti-angiogenic factors [e.g., Endostatin (Wu T. et al., 2020)]. In addition, as we all know, the combination of β-catenin and TCF4 has a low KD value and a large interaction surface. It is challenging to use a single low molecular weight inhibitor to inhibit this interaction. Hence, there is no small molecule capable of directly inhibiting Wnt signal transduction through the β-catenin target (Cui et al., 2018).

However, research has illuminated the potential of various Chinese medicines to target and modulate this pathway, offering therapeutic benefits at different stages of breast cancer. For instance, such as Ganoderma lucidum (Leyss.ex Fr.) Karst (Ganoderma lucidum) (Zhang, 2017) and metabolites of TCM [Deguelin (Murillo et al., 2009), Gigantol (Yu et al., 2018), and Chlorogenic acid (Xue et al., 2023)] have been identified to influence the upstream targets of the Wnt/β-catenin pathway, including Frizzled 7 and LRP6. By curbing the production of β-catenin, these medicines effectively stifle the Wnt/β-catenin signaling pathway. This modulation results in the suppression of breast cancer cell proliferation, invasion, and migration, while also amplifying their drug sensitivity.

In addition, the APC protein is also a key member upstream of the Wnt/β-catenin signaling pathway. APC acts as a negative regulator of β-catenin (Zhang et al., 2018) by serving as a carrier to connect glycogen synthase kinase-3β (GSK-3β) with β-catenin. This connection promotes the phosphorylation of β-catenin by GSK-3β, leading to the degradation of β-catenin and thereby maintaining β-catenin (MacDonald et al., 2009). When APC is mutated or deleted, it can still bind to β-catenin but is unable to degrade it. This results in the excessive accumulation of β-catenin in the nucleus, leading to abnormal cell proliferation, tumor formation, and drug resistance in tumor cells (Jeong et al., 2018). This mechanism explains why APC gene changes and abnormal activation of the Wnt/β-catenin signaling pathway are common in many types of cancer. It also underscores the potential of this pathway as a target for cancer treatment. It has been found that Tanshinone IIA can enhance the chemosensitivity of breast cancer cells to adriamycin by regulating the APC/β-catenin signaling pathway. It can be utilized as a potential chemosensitizer in combination with adriamycin for the treatment of breast cancer (Kanwar et al., 2010; Zhang et al., 2013; Tian et al., 2014).

Moreover, various TCM metabolites have been identified to directly target the β-catenin protein, effectively inhibiting the proliferation and metastasis of breast cancer cells and inducing apoptosis. Notable examples include Astragalus polysaccharide (Yang S. et al., 2020), Capsaicin (Wu D. et al., 2020), and Jatrophizine (Zhao and Wang, 2023), all of which are known to reduce β-catenin expression levels. Additionally, the root extract of Actinidia chinensis Planch. Gan et al. (2021) has been found to inhibit the phosphorylation of β-catenin. Psoralen (Wang et al., 2018b), the metabolite of Cullen corylifolium (L.) Medik. (Fabaceae; Psoraleae fructus), is effective in suppressing the transcriptional activity of β-catenin and Wnt target genes in breast cancer cells. These findings highlight the potential of TCM metabolites in offering targeted therapeutic actions against key pathways in breast cancer.

Lastly, TCM monomers such as Wolfiporia cocos (F.A. Wolf) Ryvarden and Gilb (Poria cocos) (Jiang and Fan, 2020) and Auriculariaauricula (L.cxHook.) Underw (Trametes robiniophila murr) (Zhang et al., 2010) as well as TCM metabolites such as Chalcone (Bortolotto et al., 2017) and Matrine (Xiao et al., 2018) have been found to target the downstream genes of the Wnt/β-catenin signaling pathway, like Cyclin D1, c-myc, and Bcl-2. Their action effectively disrupts the cell cycle progression in breast cancer cells, halting their proliferation and triggering apoptosis. This comprehensive understanding of the interplay between TCM and the Wnt/β-catenin pathway underscores the profound potential of TCM in breast cancer therapeutics (Figure 2C).

## 4 Heterogeneity and unique characteristics of Wnt/β-catenin activated breast cancer

Breast cancer stands out as the most prevalent malignant tumor among females. A closer look at breast cancer cells reveals a complex landscape: mutations differ from one patient to another, and within a single patient’s breast cancer tissue, a myriad of lesions can be identified. This complexity is further underscored by the varied expression levels of pivotal proteins within the same tissue, such as the Estrogen Receptor (ER), Progesterone Receptor (PR), and Human Epidermal Receptor 2 (HER-2) (Visvader, 2011; Turashvili and Brogi, 2017). Such variations underscore the pronounced heterogeneity inherent to breast cancer. This heterogeneity manifests in the form of multiple breast cancer subtypes. Clinically, two predominant subtypes emerge based on receptor status: the ER-positive, PR-positive, HER-2 amplification type, and the Triple-Negative Breast Cancer (TNBC) (Xu et al., 2022). Delving deeper into molecular markers, breast cancer can be categorized into Luminal A type, Luminal B type, HER-2 overexpression type, and Triple-Negative type, determined by the expression levels of ER, PR, HER-2, and Ki-67 (Gangrade et al., 2018; Fumagalli and Barberis, 2021; Liu H. Y. et al., 2022). It is crucial to note that the prognosis varies across different clinical stages, molecular types, and pathological types of breast cancer. Even among patients with identical clinical stages, outcomes can diverge significantly based on molecular and pathological distinctions (Fumagalli and Barberis, 2021). Given this complexity, there’s a pressing need for individualized treatment strategies tailored to specific breast cancer subtypes.

**FIGURE 3:**
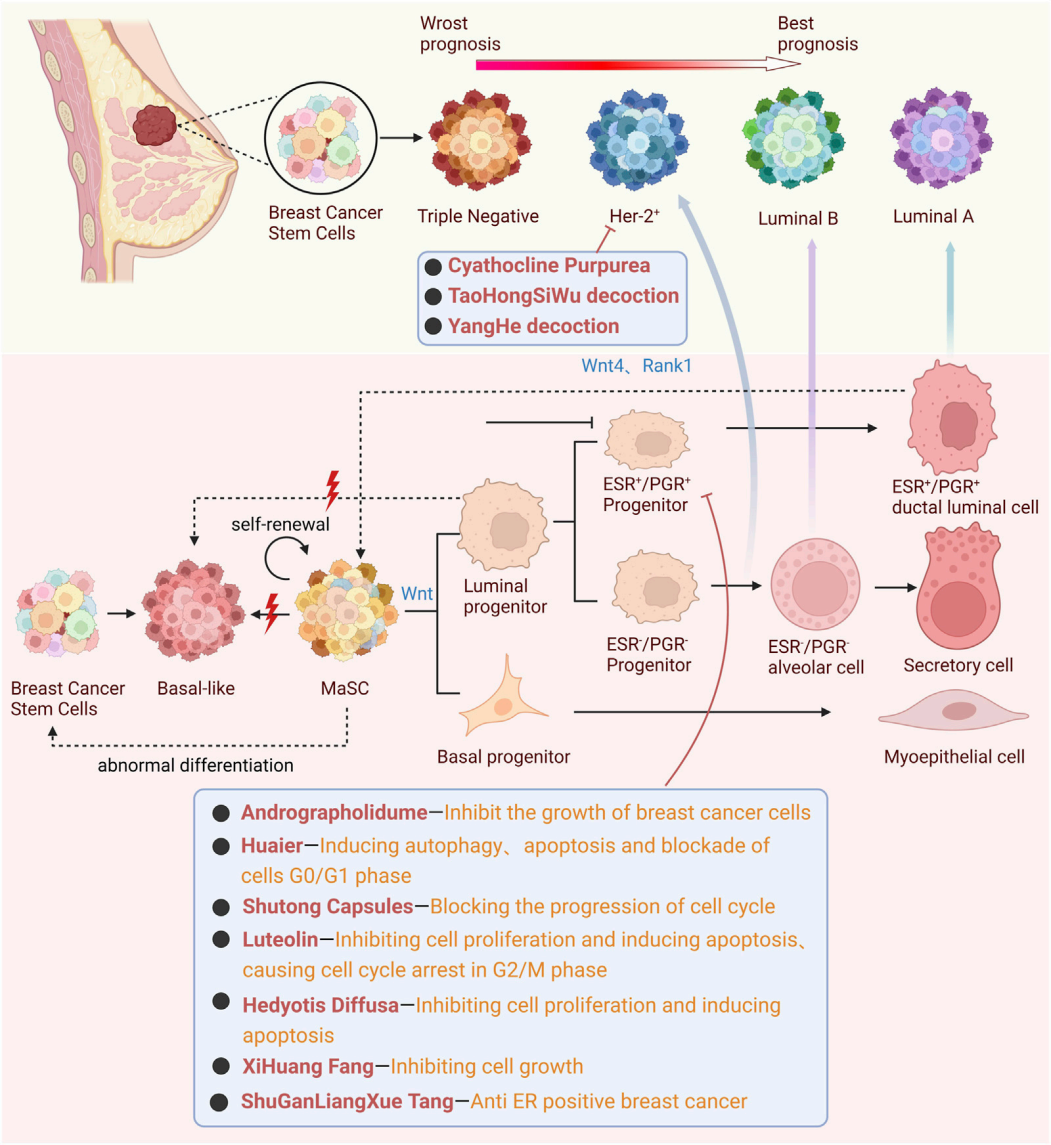
Mechanistic interplay of the Wnt signaling pathway in breast cancer heterogeneity: TCM’s targeting approach for HER-2^+^ and ER^+^ Subtypes.

In clinical practice, the treatment approach for breast cancer varies based on the cancer’s receptor status. Patients with ER-positive and PR-positive breast cancer often opt for selective ER modulators (Maselli et al., 2019) and aromatase inhibitors (Ratre et al., 2020) as part of their treatment regimen. For those with HER2-positive breast cancer, the treatment typically includes monoclonal antibodies (Bighin et al., 2013), tyrosine kinase inhibitors (Deeks, 2017; Dhillon, 2019), and antibody-drug conjugates (Chung et al., 2020). In contrast, triple-negative breast cancer is frequently treated with chemotherapeutic agents like Doxorubicin, Cyclophosphamide, and Vinorelbine Tartrate. However, these widely used chemotherapy drugs in clinical settings are often associated with a range of side effects, including gastrointestinal disturbances (Zhou et al., 2013; Sartaj et al., 2021a; Farghadani and Naidu, 2022), osteoporosis (Essex et al., 2019; Shapiro, 2021; Meyer et al., 2024), bone marrow suppression (Zhou et al., 2013; Koliou et al., 2018; Han et al., 2021), dyslipidemia (Duman et al., 2012), and drug resistance (Sartaj et al., 2021b; Yu S. W. et al., 2023). On the other hand, TCM has demonstrated broad applicability in various types and clinical stages of breast cancer. It offers the advantages of multi-component and multi-target comprehensive treatment, with lower toxicity compared to chemical drugs. This highlights TCM’s potential as a complementary or alternative therapeutic approach in the multifaceted treatment of breast cancer (Qi et al., 2015; Zhang et al., 2020). In light of this, TCM offers a promising avenue. Traditional Chinese Medicine’s holistic approach, with its diverse array of botanical drugs, is uniquely positioned to address the multifaceted nature of breast cancer. By targeting specific pathways and mechanisms, such as the Wnt/β-catenin signaling, TCM provides a nuanced approach that aligns with the heterogeneity of breast cancer, holding promise for more effective and tailored therapeutic outcomes (Raut et al., 2022) (Figure 3).

### 4.1 ER-positive breast cancer

ER-positive breast cancers make up roughly 70% of all breast cancer cases (Zhu et al., 2021). The presence of ERα stands as a pivotal prognostic marker and plays a decisive role in determining clinical outcomes for ER-positive breast cancer patients (Li et al., 2014). This makes it a promising therapeutic target. Consequently, the discovery of selective ER modulators and selective estrogen downregulation factors is of paramount importance for treating this subtype of breast cancer (Park and Jordan, 2002). Commonly prescribed endocrine drugs in clinical settings include tamoxifen, anastrozole, and fulvestron (Bross et al., 2003). While these drugs have shown potential in improving progression-free survival rates (Robertson et al., 2016; Maass et al., 2019), they come with their set of challenges, including notable side effects and the potential to induce drug resistance (Qi et al., 2016). Furthermore, their effectiveness in enhancing overall survival rates and progression-free survival for advanced ER-positive patients remains somewhat constrained (Slamon et al., 2020; Albanell et al., 2022).

In this context, TCM offers a beacon of hope. With its minimal side effects, TCM presents a promising avenue for breast cancer management (Zhao M. H. et al., 2022). For instance, The *ShuTong* Capsule plays a role in ERα positive breast cancer cell lines MCF7 and T47D by down-regulating Cyclin D1 and ERα, the downstream target genes of the Wnt/β-catenin signaling pathway, thereby inhibiting breast cancer cell cycle progression (Du et al., 2014). Meanwhile, Scleromitrion diffusum (Willd.) R.J.Wang [Rubiaceae; Herba hedyoti diffusae] (Li et al., 2017), *XiHuang* Formula (Hao et al., 2018), and *ShuGanLiangXue* Decoction (Zhou et al., 2014) have been shown to have anti-tumor effects in ER-positive breast cancer. Furthermore, certain metabolites derived from TCM have been shown to inhibit the proliferation of ER-positive breast cancer cells and induce apoptosis simultaneously, such as andrographolide, the primary active metabolite of Andrographis paniculata (Burm.f.) Wall. ex Nees (Acanthaceae; Andrographis herba), in nude mice bearing breast cancer xenografts with MCF-7 cells (ip, 150 mg/kg/day, 16 days), can hinders Estrogen Receptor 1 transcription by targeting the ROS-FOXM1 axis and indirectly curtails the Wnt/β-catenin signaling pathway, thus stalling breast cancer cell proliferation (Xu et al., 2022). Research by Qi et al. (2016) highlighted that extracts derived from Auriculariaauricula (L.cxHook.)Underw (Trametes robiniophila murr), in female mice bearing breast cancer xenografts with MCF-7 cells (ig, 100 μL solution containing 50 mg, 40 days), inhibit the Wnt/β-catenin signaling pathway by targeting the AKT/mTOR pathway, leading to cell cycle blockade at the G0/G1 phase, and triggering autophagy-induced apoptosis. Luteolin, which is found in various vegetables, in ER-positive breast cancer cell lines MCF7, indirectly suppresses the Wnt/β-catenin signaling pathway by inhibiting the PI3K/AKT/mTOR signaling pathway, resulting in cell cycle arrest at the G2/M phase, reduced cell proliferation, and the initiation of apoptosis (Wu H. T. et al., 2020).

### 4.2 PR-positive breast cancer

PR, functioning as a regulatory gene for estrogen and ER (Li et al., 2022a), stands as a pivotal prognostic biomarker, influencing both the overall survival rate and disease-free survival rate in breast cancer patients (Mohammed et al., 2015). Intriguingly, Rank1 and Wnt4, direct targets of PR, play an indispensable role in paracrine actions, particularly in the induction of mitogenic signals by PR (Fu N. Y. et al., 2020). One of the remarkable capabilities of PR lumen cells is their ability to produce Wnt4. This, in turn, promotes the self-renewal of mammary stem cells (MaSCs) via the Wnt/β-catenin signaling pathway. This observation finds support in the research by Cai et al. (2014), where they identified the Wnt receptor agonist R-sport 1 as a potential novel conduit for transmitting ovarian hormone signals directly to MaSCs.

In a clinical setting, PR-positive breast cancer patients typically lean towards a combined treatment strategy, integrating endocrine therapy with adjuvant therapy, aiming to preemptively counteract cancer’s progression (Li Z. et al., 2022). However, the interplay between TCM and PR-positive breast cancer remains an area yet to be fully explored, highlighting the need for more in-depth research in this domain.

### 4.3 HER-2 positive breast cancer

HER-2 positive breast cancer is categorized into two specific subtypes: Luminal B and HER-2 overexpression (Zeng and Yang, 2017). Currently, the primary pharmacological interventions for HER-2 positive breast cancer include anthracyclines, paclitaxel (Schneeweiss et al., 2015), aromatase inhibitors (Ellis et al., 2001), and other related medications. However, a significant concern arises from the pronounced adverse reactions and potential drug resistance associated with most chemotherapy drugs. These side effects can range from gastrointestinal issues and immune system disruptions to cardiotoxicity and other complications (Gonzalez-Angulo et al., 2007; Zeng and Yang, 2017). Given these challenges, the standalone therapeutic efficacy of chemotherapy drugs for HER-2 positive breast cancer remains less than optimal (Javir et al., 2020).

In response to these challenges, the medical community has broadened its approach to breast cancer management. Complementary and Alternative Medicine (CAM) has gained traction as a viable strategy for both prevention and treatment (Zeng and Yang, 2017). Within the CAM spectrum, Chinese medicine stands out, offering a rich history and a plethora of therapeutic options for breast cancer management. For instance, cyathocline purpurea (Buch.-Ham. ex D. Don) Kuntze (Asteraceae; Cyathoclines purpureae herba), in HER-2 positive MDA-MB-453 cell line, showcases its therapeutic potential by reducing the size of MDA-MB-231 cells, inhibiting their proliferation and movement, and initiating their apoptosis. This is achieved through its influence on the epithelial-mesenchymal transition (EMT), tumor necrosis factor-alpha (TNF-α), and the Wnt/β-catenin signaling pathways (Javir et al., 2020). Another notable traditional remedy, *TaoHongSiWu* decoction, has demonstrated its capability to inhibit the Wnt/β-catenin signaling pathway by targeting the PI3K/AKT pathway, effectively curtailing the proliferation and metastasis of breast cancer cells (Jiang et al., 2021). Furthermore, research spearheaded by Zeng and his colleagues has highlighted the therapeutic potential of *YangHe* decoction specifically for HER-2 positive breast cancer (Zeng and Yang, 2017).

### 4.4 Triple-negative breast cancer

Triple-negative breast cancer, characterized by the absence of ER, PR, and HER-2 expressions (Buyuk et al., 2022), constitutes 15%–20% of all breast cancer cases (Yao et al., 2017). Although the incidence rate is low, it has a very high mortality rate (Solzak et al., 2017). The lack of hormone receptors and the non-overexpression of HER-2 protein render molecular targeting and endocrine therapies particularly challenging for TNBC (Yang C. Q. et al., 2020; Sun et al., 2020; Li Y. P. et al., 2022; Lu et al., 2023). Compounding the challenge, TNBC’s aggressive nature results in a higher risk of distant metastasis, leading to a more dire prognosis compared to other breast cancer subtypes (Saranya et al., 2020; Yang et al., 2021). In the clinical realm, the primary therapeutic interventions for advanced TNBC remain anthracycline chemotherapy drugs (Coates et al., 2015; Yu et al., 2020; Zhao et al., 2021). While these can extend patient survival, their pronounced toxic effects often prove intolerable for many patients (Goto et al., 2018). Given this backdrop, the quest for efficacious treatments for TNBC with minimal side effects has intensified.

Recent years have witnessed a surge in research exploring the potential anti-tumor properties of TCM, particularly its ability to modulate the body’s signaling pathways. These studies hint at the promise of TCM as a novel therapeutic avenue for TNBC (Yang et al., 2021). Whether as a sustained supplementary treatment or a potential alternative, TCM is emerging as a beacon of hope in the battle against TNBC. For instance, tannins, the primary active metabolite of Syzygium guineense (Willd.) DC. [Myrtaceae; Syzygium guineense] reduces the stability and transcriptional activity of β-catenin by inhibiting Wnt3a, thereby preventing the proliferation of BT-20 cells (Koval et al., 2018). Jatrophone, the primary active metabolite of Jatropha gossypiifolia L. [Euphorbiaceae; Jatropha gossypiifolia], can decrease the steady-state and non-phosphorylated (activated) β-catenin levels. It directly inhibits the migration of triple-negative breast cancer cells by targeting the cancer-causing Wnt10b/β-catenin signaling pathway. This botanical drug is anticipated to emerge as a potent new chemotherapeutic agent for treating triple-negative breast cancer, which is known for its high resistance to chemotherapy (Fatima et al., 2017). (Table 1).

**TABLE 1:** Active phytoconstituents of TCM approaches targeting the Wnt/β-catenin pathway in TNBC treatment.

Plant metabolite	Source	Molecular formula	Extraction method	Cell	Animal	Administration mode	Dose	Course of treatment	Contrast mode	Function	Mechanism	References
Cardamonin	*Alpinia hainanensis* K.Schum. [Zingiberaceae; Alpiniae katsumadai semen]	C_16_H_14_O_4_	—	MDA-MB-231 cells	Mice	ip	3 mg/kg/day	4 weeks	5-Fu (positive control) and normal saline (negative control)	Blocking EMT and cell invasion	Stability and nuclear translocation of β-catenin ↓,β-catenin target gene ↓	Shrivastava et al. (2017), Jin et al. (2019a)
				MCF-7,MDA-MB-231, and BT-549 cells			2.5 and 5 mg/kg/day	25 days	Normal saline (negative control)			
Crocin	*Crocus sativus* L. [Iridaceae; Croci stigma]	C_44_H_64_O_24_	—	MDA-MB 231 cells	—	—	—	—	Doxorubicin (positive control)	Inhibition of cell metastasis	Wnt/β-catenin↓	Arzi et al. (2018), Farahi et al. (2021), Chavoshi et al. (2023)
			Extraction from Iranian saffron powder using active column chromatography	4T1 cells	Mice		200 mg/kg, thrice per week	4 weeks	Metformin (positive control) and normal mice (no-treatment control)			
			Repeated extraction of Iranian saffron powder with n-hexane	—					Normal mice (no-treatment control)			
Saikosaponin D	*Bupleurum chinense* DC. [Apiaceae; Bupleuri radix]	C_42_H_68_O_13_	Ethanol extraction, EtOH/H_2_O elution followed by separation and preparation using C18ME chromatography column	HCC1937,MDA-MB-468 and MDA-MB-231 cells	—	—	—	—	Paclitaxel (positive control)	Inhibiting cell proliferation and inducing apoptosis	β-catenin and downstream target genes ↓	Wang et al. (2018a), Fu et al. (2020b)
			—	MDA-MB-231 cells								
Schisandrin A	Schisandra chinensis (Turcz.) Baill. [Schisandraceae; Schisandrae chinensis fructus]	C_24_H_32_O_6_	—	MDA-MB-231,BT-549 and MCF-7 cells	Mice	po	25 mg/kg/day	12 days	Ethoxylated castor oil (negative control)	Inducing cell cycle arrest and apoptosis	Adjusting Wnt/ER stress signaling path	Xu et al. (2019)
Gomisin M2	Schisandra arisanensis subsp. viridis (A.C.Sm.) R.M.K.Saunders [Schisandraceae; Schisandra viridisA.C.smith]	C_22_H_26_O_6_	—	MDA-MB-231 and HCC106 cells	Zebrafish	—	—	—	Doxorubicin (positive control) and DMSO (negative control)	Inhibiting cell proliferation	Wnt/β-catenin↓	Yang et al. (2019)
Gugulipid	*Commiphora wightii* (Arn.) Bhandari [Burseraceae; *Commiphora myrrha*]	C_21_H_28_O_2_	—	MDA-MB-231 cells	—	—	—	—	DMSO (negative control)	Inhibiting cell proliferation and inducing cell apoptosis	β-catenin/TCF-4↓	Jiang et al. (2013)
Yuanhuacine	Daphne genkwa Siebold & Zucc. [Thymelaeaceae; Genkwa flos]	C_37_H_44_O_10_	MeOH-H_2_O crude extraction, vacuum liquid chromatography and high performance liquid chromatography separation	HCC1806, HCC70 cells	Mice	ip	20 mg/kg/2 days	4 days	Paclitaxel (positive control) and DMSO (negative control)	Promote the expression of anti-tumor cytokines and have an effective anti-tumor effect *in vivo*	Activate PKC and induce THP-1 differentiation	Fermaintt et al. (2021)
Luteolin	Carrots *Daucus carota* L. [Apiaceae; Daucus carota var. sativa hoffm] and *Perilla frutescens* (L.) Britton [Lamiaceae; Perillae folium]	C_15_H_10_O_6_	—	MCF-7 cells	—	—	—	—	LY294002, MK-2206, and rapamycin (positive control) and DMSO (negative control)	Inhibit cell proliferation and metastasis	Inhibition of AKT/mTOR signaling pathway,β-catenin↓, MMP9↓	Lin et al. (2017), Wu et al. (2020c), Wu et al. (2021a)
				MDA-MB-231, MDA-MB-486, 4T1 and BT-549 cells					MK-2206, rapamycin (positive control) and DMSO (negative control)			
				MDA-MB-231and BT-549 cells	Mice	ip	100 mg/kg, thrice per week	8 weeks	PBS (negative control)			

### 4.5 TCM interventions targeting breast cancer stem cells

Currently, the cancer stem cell model is primarily used to explain the heterogeneity of breast cancer as described above. This theory posits that cell diversity and tumor grade are generated by breast cancer stem cells (BCSCs) that can form transplantable tumors and rebuild tumor heterogeneity (Rafii and Lyden, 2003; Vermeulen et al., 2008). These cells play a crucial role in influencing early metastasis (Bozorgi et al., 2015; O’Conor et al., 2018), drug resistance (Moreira et al., 2018; Park et al., 2019), and the overall prognosis of the disease. At the same time, some molecular markers, such as CD44^+^/CD24^-/low^ (Al-Hajj et al., 2003; Sheridan et al., 2006), ALDH1^+^ (Ginestier et al., 2007; Morimoto et al., 2009), CD133 (Kim et al., 2015), CD61 (Vaillant et al., 2008), CD49f (Ye et al., 2017), and CXCR4 (Ablett et al., 2014; Trautmann et al., 2014), can be used as biomarkers to identify BCSCs, and may serve as therapeutic targets for small molecular inhibitors. Therefore, many researchers realize that targeting BCSCs is crucial to achieve long-lasting remission of breast cancer (Gwynne et al., 2021). In experimental research, the common and unique characteristics of normal stem cells and cancer stem cells have been utilized to develop robust stem cell models. The commonly used model for BCSCs is 3D multicellular stem-like spheroids. Compared with 2D monolayer culture, 3D culture exhibits remarkable tumorigenicity, better simulating *in vivo* behavior, facilitating mechanism research, and aiding in the development of targeted drugs (Antoni et al., 2015).

In addition, regulatory signaling pathways targeting BCSCs have been developed for the treatment of breast cancer, especially in cases of therapeutic resistance (Zhou et al., 2019). The Wnt/β-catenin signaling pathway, as the primary driving force behind the onset and progression of breast cancer (Qayoom et al., 2021; Mao X. D. et al., 2022), plays a crucial role in sustaining the activity of BCSCs, facilitating tumor metastasis, and triggering the expression of drug-resistant genes (Lin et al., 2018; Ke et al., 2022). Based on these insights, inhibiting the activity of BCSCs by regulating the Wnt/β-catenin signaling pathway has gradually become the main focus of research (Feng et al., 2023). In recent years, an increasing number of studies have found that many metabolites of traditional Chinese medicine and compounds from traditional Chinese medicine align with this treatment concept, as detailed in (Table 2).

**TABLE 2:** Active phytoconstituents of TCM interventions targeting BCSCs.

Medicine	Source/composition	Structural formula	Extraction method	Cell	Animal	Administration mode	Dose	Course of treatment	Contrast mode	Effect on BCSCs	Mechanism	References
Curcumin	*Curcuma longa* L. [Zingiberaceae; *Curcumae longae* rhizoma]	C₂₁H₂₀O₆	—	SUM159 and MCF-7 cells	—	—	—	—	—	Inhibit its proliferation, induce its apoptosis, and effectively reduce its activity; Reduce the resistance of BCSCs to mitomycin C; Inhibition of Shh and Wnt/-catenin pathways	Shh and Wnt/β-catenin pathways ↓, Regulating Bcl-2 family	Mukherjee et al. (2014), Zhou et al. (2017), Li et al. (2018c)
				MCF-7 and T47D cells								
				MDA-MB-231 and MCF-7 cells					Mitomycin C (positive control)			
				MDA-MB-231 cells					—			
				MCF-7 cells								
Tanshinone ⅡA	*Salvia miltiorrhiza* Bunge [Lamiaceae; Salviae miltiorrhizae radix et rhizoma]	C_19_H_18_O_3_	—	MCF-7 cells	—	—	—	—	Doxorubicin (positive control)	Eliminate cancer cells including BCSCs; Inhibit the formation of breast nodules; Enhance the chemotherapy sensitivity of breast cancer; Promote the apoptosis of BCSCs	Excretion of ABC transporter ↓, Regulating IL-6/STAT3/NF-κB signal pathway	Lin et al. (2013), Li and Lai (2017), Kim et al. (2019), Zhao et al. (2022a)
				MCF-7 and MDA-MB-231 cells	Mice	mammary fat pad injection	10 mg/kg/week	13 weeks	Mice not receiving chemotherapy (negative control)			
				MCF-7 cells		ip	10, 20, and 40 mg/kg, thrice per week	4 weeks	Normal saline (negative control)			
*Angelica sinensis*	*Angelica sinensis* (Oliv.) Diels [Apiaceae; Angelicae Sinensis radix longae rhizoma]	C_12_H_14_O_2_	Distilled water is boiled and filtered, concentrated by rotary evaporator, and the filtrate is freeze-dried into powder	MCF-7 and MDA-MB-231 cells	Mice	po	2.5 g/kg/day	22 days	Tamoxifen (positive control) and normal saline (negative control)	Promoting the activity of tumor stem cells in ER-positive breast cancer	Expression of ERαwas induced *in vivo* and *in vitro*	Zhu et al. (2021)
Bufalin	Bufonis venenum	C_24_H_34_O_4_	—	MDA-MB-231 and HCC-1937cells	Mice	—	1 mg/kg, thrice per week	2 weeks	DMSO (negative control)	Reduce the stem cell characteristics of TNBC stem cells and inhibit their proliferation	SOX2/OCT4 Axis Mediated	Chen et al. (2020)
Esculentoside A	*Phytolacca acinosa* Roxb. [Phytolaccaceae; Phytolaccae radix]	C_42_H_66_O_16_	—	Mouse breast cancer cells EMT6 strain and MCF-7 cells	Mice	ig	10, 20, and 40 mg/kg, five times a week	6 weeks	Normal saline (negative control)	Inhibited the proliferation of BCSCS and the formation of breast balls; Inducing apoptosis of breast stem cells	IL-6/STAT3 signal pathway↓	Liu et al. (2018)
Silibinin	Silybum marianum (L.) Gaertn. [Asteraceae; Silybi fructus]	C_25_H_22_O_10_	—	MDA-MB-468 cells	—	—	—	—	—	Inhibit the growth of tumor cells	Reducing dryness and inducing apoptosis	Abdollahi et al. (2015), Firouzi et al. (2022)
				MCF-7, MDA-MB-231,MDA-MB-468 and 4T1 cells	Mice		1.2 mg/kg/72 h	20 days	Ethanol (negative control)			
Pterostilbene	Blueberries or Vitis vinifera L. [Vitaceae; Vitis viniferae fructus]	C_16_H_16_O_3_	—	MCF-7, MDA-MB-231, MDA-MB-468 and 4T1 cells	Mice	ig	50 mg/kg/day	29 days	CMC-Na (negative control), cisplatin (positive control) and normal mice (no-treatment control)	Reduce the dryness of BCSCs; Induce cell necrosis in BCSCs such as membrane damage and bubble formation; Inhibit the formation of milk globules	GSK3β signal transduction,β-catenin phosphorylation,c-myc, cyclin D1↓	Wu et al. (2015), Ma et al. (2022)
				MCF-7 cells	—	—	—	—	Paclitaxel (positive control)			
Resveratrol	Vitis vinifera L. [Vitaceae; Vitis Viniferae fructus], and *Arachis hypogaea* L. [Fabaceae; *Arachis hypogaea* linn]	C_14_H_12_O_3_	—	MCF-7 and SUM159 cells	Mice	iv	100 mg/kg/day	2 weeks	normal saline (negative control)	Induce autophagy and inhibit the invasion of BCSCs	Wnt/β-catenin↓, Tumor suppressor miRNAs↑	Hagiwara et al. (2012), Fu et al. (2014)
				MDA-MB-231 cells		ip	25 mg/kg/day					
Matrine	Sophora flavescens Aiton [Fabaceae; Sophorae flavescentis radix]	C_15_H_24_N_2_O	—	MCF-7 and T47-D cells	Mice	ip	20 mg/kg/day	4 weeks	Cisplatin (positive control)	Inhibiting the differentiation and self-renewal of BCSCs	Lin28A↓	Li et al. (2020)
*AiDuQing*	Scleromitrion diffusum (Willd.) R.J.Wang [Rubiaceae; Herba hedyoti diffusae], *Curcuma zedoaria* (Christm.) Roscoe [Zingiberaceae; Curcumae rhizoma], Astragalus mongholicus Bunge [Fabaceae; Astragali radix] and Glycyrrhiza uralensis Fisch. ex DC. [Fabaceae; Glycyrrhizae radix et rhizoma]	—	Extract with ethanol, concentrating and freeze-dry	MCF-7 and MDA-MB-231 cells	Mice, Zebrafish	ig	100 mg/kg/day	—	Paclitaxel (positive control) and normal saline (negative control)	Inhibit the self-renewal, Differentiation and Autophagy of BCSCs, Inhibit its resistance to paclitaxel	Mediated by GRP78/β-catenin/ABCG2 signal transduction	Liao et al. (2021)
*SiNiSan*	Bupleurum chinense DC. [Apiaceae; Bupleuri radix], Paeonia lactiflora Pall. [Paeoniaceae; Paeoniae radix alba], Citrus × aurantium L. [Rutaceae; Aurantii fructus Immaturus] and Glycyrrhiza uralensis Fisch. ex DC. [Fabaceae; Glycyrrhizde radix et rhizoma]	—	Reflux at 95°C, concentrate by rotary evaporation at 65°C and freeze-dry	4T1 cells	Mice	po	1.65 g/kg/day	4 weeks	—	Inhibition of lung metastasis induced by chronic psychological stress and stem cell nature of breast cancer cells	Inhibition of Wnt/β-catenin signal transduction	Zheng et al. (2021)

For example, naringenin, an active metabolite found in tomatoes, grapefruits, and oranges, can inhibit the proliferation and migration, and induce apoptosis of BCSCs by regulating the transforming growth factor-β (TGF-β) and Wnt/β-catenin pathways in MCF-7 cells. It is expected to become a targeted drug for BCSCs (Hermawan et al., 2021). Quercetin, a metabolite found in various fruits, vegetables, nuts, and seeds, has been shown to inhibit the proliferation, self-renewal, and invasiveness of BCSCs in a mouse model carrying MCF-7 cells. This inhibition is achieved by suppressing the PI3K/Akt/mTOR signaling pathway, which is related to the Wnt/β-catenin signaling pathway, and by reducing the expression levels of proteins associated with cancer progression (Li X. L. et al., 2018; Wang R. et al., 2018). At the same time, it has been found that quercetin can reverse the multidrug resistance of breast cancer cells by down-regulating the expression of P-gp and eliminating cancer stem cells through YB-1 nuclear translocation (Li S. Z. et al., 2018; Wang S. Y. et al., 2023).

## 5 TCM compounds and their role in modulating the Wnt/β-catenin pathway in breast cancer treatment

Traditional Chinese Medicine, deeply rooted in China’s rich cultural heritage, stands out for its unique diagnostic and therapeutic approaches, coupled with its proven clinical efficacy. Beyond its intrinsic value, TCM offers a complementary approach to conventional cancer treatments. It can amplify the anti-tumor effects of standard therapies while simultaneously mitigating their side effects. When combined with chemotherapy and radiotherapy, TCM can alleviate tumor-induced symptoms, such as cancer pain, and has been observed to prolong the survival of patients, especially those in advanced stages post-surgery (Li et al., 2012).

A growing body of research underscores the potential of TCM monomers in breast cancer treatment. These compounds have demonstrated their ability to disrupt the cell cycle, inhibit cell proliferation and growth, curtail cell migration and invasion, and trigger apoptosis. Intriguingly, many of these effects are mediated through the modulation of the Wnt/β-catenin signaling pathway, positioning TCM as a promising avenue in the therapeutic landscape of breast cancer.

### 5.1 Cell cycle progression modulation

Traditional Chinese Medicine monomer and its metaboliteshave shown significant potential in modulating the cell cycle progression of breast cancer cells, primarily through their interaction with the Wnt/β-catenin signaling pathway. Auriculariaauricula (L.cxHook.)Underw (Trametes robiniophila murr) (Zhang et al., 2010; Ding et al., 2016; Yang L. et al., 2017; Gao et al., 2017) and Nobiletin (Wu Y. et al., 2021), the latter being a major metabolite of Citrus folium, in MCF-7 and MDA-MB-231 cells, exhibit the capability to induce G0/G1 cell cycle arrest in breast cancer cells. This is attributed to their inhibitory effect on Cyclin D1 expression, a key downstream target of the Wnt/β-catenin pathway. Similarly, Chalcones, derived from Glycyrrhiza uralensis Fisch. ex DC. (Fabaceae; Glycyrrhizae radix et rhizoma), can halt the MCF-7 breast cancer cell cycle at the G1 phase by downregulating the expression of Bcl-2 and Cyclin D1, both of which are integral to the Wnt/β-catenin pathway (Bortolotto et al., 2017).

Psoralen, the metabolite of *Cullen corylifolium* (L.) Medik. (Fabaceae; Psoraleae fructus), on the other hand, impacts the Wnt/β-catenin signaling pathway by affecting the cytoplasmic accumulation and nuclear translocation of β-catenin. This results in diminished overall β-catenin levels. Moreover, Psoralen can arrest the G0/G1 phase in MCF-7 breast cancer cells and the G2/M phase in MDA-MB-231 cells (Wang et al., 2018d). Capsaicin, the primary spicy metabolite in *Capsicum annuum* L. (Solanaceae; Capsici fructus), has also shown promise. A study by Wu et al. (2020) revealed its potential to induce G2/M cell cycle arrest in MDA-MB-231 cells. This is achieved through a multifaceted mechanism involving reduced CDK8 expression, diminished phosphorylation of PIK3 and AKT, and the downregulation of Wnt β-catenin expression (Wu D. et al., 2020).

### 5.2 Inhibition of cell proliferation and growth

Through their interactions with the Wnt/β-catenin signaling pathway, TCM monomer and its metabolites have demonstrated remarkable efficacy in regulating the growth and proliferation of breast cancer cells. Ganoderma lucidum (Leyss.ex Fr.) Karst (Ganoderma lucidum) has been observed to inhibit the proliferation of human MDA-MB-231 and mouse 4T1 breast cancer cells. This is achieved by reducing LRP6 phosphorylation and inhibiting the expression of Axing, a Wnt target gene activated by Wnt3. Furthermore, Ganoderma lucidum (Leyss.ex Fr.) Karst (Ganoderma lucidum) has been shown to effectively suppress the Wnt/β-catenin signaling pathway. The concentration of Ganoderma lucidum (Leyss.ex Fr.) Karst (Ganoderma lucidum) required to achieve these effects is consistent with the concentration needed to inhibit breast cancer cell proliferation and migration (Zhang, 2017).

Deguelin, on the other hand, in MDA-MB-231 cells can reduces the expression of β-catenin and its downstream target gene, c-myc, by downregulating frizzled 7 expression. This action has been linked to the inhibition of TMBC cell proliferation (Murillo et al., 2009; Rodenberg and Brown, 2009). *Actinidia chinensis Planch.* root extract (AcRoots) impacts the proliferation of breast cancer MDA-MB-231 and MDA-MB-453 cells by decreasing levels of phosphorylated AKT, phosphorylated GSK-3β, and β-catenin (Gan et al., 2021). Ophiopogonin D, a steroidal glycoside derived from the TCM Ophiopogon japonicus (Thunb.) Ker Gawl. (Asparagaceae; Ophiopogonis radix), has been observed to dose-dependently inhibit the upregulation of the Wnt/β-catenin signaling pathway in MDA-MB-231 cells (Zhu et al., 2020). Honokiol is a bioactive bisphenol botanical drug found in the leaves and extracts of Magnolia officinalis Rehder and E.H.Wilson (Magnoliaceae; Magnoliae officinalis cortex) (Banik et al., 2019), which inhibits the growth of breast cancer cells and the proliferation of SK-BR-3 cells in diet-induced obese mouse models (ip, 3 mg/day, three times per week, 4 weeks) by inhibiting Wnt/β-catenin signaling pathway (Avtanski et al., 2015; Shi et al., 2020).

### 5.3 Modulation of cell migration and invasion

Betulinic acid, a metabolite extracted from Betula pendula subsp. mandshurica (Regel) Ashburner and McAll. (Betulaceae; Betula platyphylla suk), has been identified to elevate the expression of GRP78, curtail β-catenin-driven aerobic glycolysis in cell cultures, and inhibit lung metastasis of breast cancer cells in nude mice carrying MDA-MB-231 cells (ip, 125 and 250 mg/kg/day, 4 weeks) by regulating GRP78/β-catenin/c-Myc signal cascade. (Zheng Y. F. et al., 2019). Astragaloside IV, a primary triterpenoid from Astragalus mongholicus Bunge (Fabaceae; Astragali radix), operates by reducing Rac1 levels via Vav3 downregulation. This action subsequently affects β-catenin nuclear expression, thereby curbing the invasive tendencies of MDA-MB-231 breast cancer cells (Jiang et al., 2017). Lastly, Telocinobufagin, a metabolite from Bufonis venenum, indirectly interacts with the Wnt/β-catenin signaling pathway by regulating the PI3K/AKT/ERK/snail signaling cascade. This regulation results in a notable decrease in the mobility and invasiveness of breast cancer cells in nude mice carrying 4T1 cells (ip, 20 µg, three times a week, for 2 weeks) (Gao et al., 2018).

The LRP6 receptor, a type I transmembrane protein, is part of the low density lipoprotein receptor gene family and is highly conserved. Its phosphorylation is often seen as an indication of Wnt/β-catenin signaling pathway activation (Pinson et al., 2000; Tamai et al., 2000; Wehrli et al., 2000; Jeong and Jho, 2021). Gigantol, a hydroxybenzene metabolite sourced from several medicinal orchids, has been identified to suppress the Wnt/β-catenin signaling pathway by reducing the levels of phosphorylated LRP6 and cytoplasmic β-catenin. Consequently, it significantly curtails the survival and migratory capabilities of breast cancer HEK293, MDA-MB-231 and MDA-MB-468 cells (Yu et al., 2018). Similarly, Ganoderma lucidum (Leyss.ex Fr.) Karst (Ganoderma lucidum) has been observed to inhibit the migration of breast cancer cells, specifically MDA-MB-231 and 4T1 cellls (Zhang, 2017). Chlorogenic Acid, a notable bioactive metabolite present in various TCMs like *Lonicera japonica* Thunb. (Caprifoliaceae; Lonicerae japonicae flos) and Eucommia ulmoides Oliv. (Eucommiaceae; Eucommiae cortex), directly interacts with the Wnt/β-catenin signaling co-receptor LRP6. This interaction leads to a reduction in the expression of LRP6, p-LRP6, and β-catenin in MCF-7 breast cancer cells, subsequently inhibiting the invasion of these cells (Xue et al., 2023).

Furthermore, Jatrorrhizine, the metabolite of Coptis chinensis Franch. (Ranunculaceae; Coptidis rhizoma) has been found to induce apoptosis of breast cancer cells in nude mice bearing 4T1 cells (ip, 2.5 and 5 mg/kg, 4 weeks) and impede their metastasis in a dose-dependent manner through the modulation of the Wnt/β-catenin signaling pathway (Sun et al., 2019; Zhao and Wang, 2023). Astragalus polysaccharide has been found to exhibit a significant inhibitory effect on the migration and invasion of MDA-MB-231 cell transplanted tumor model in nude mice (ig, 100 mg/kg/day, 15 days). This effect is dose-dependent and is achieved through the downregulation of the Wnt/β-catenin signaling pathway, as well as the downregulation of c-Myc and Cyclin D1 levels, and the inhibition of EMT (Jiao et al., 2016; Ma et al., 2016; Yang S. et al., 2020). Spatholobus suberectus Dunn [Fabaceae; Spatholobi caulis] (Chen et al., 2016) and some metabolites of TCM, such as baicalein (Ma et al., 2016), emodin (Liu et al., 2020), acRoots (Gan et al., 2021), ophiopogonin D (Zhu et al., 2020), licochalcone A (Huang et al., 2019), and capsaicin (Wu D. et al., 2020) have demonstrated promising potential in the inhibition of invasive and migratory behaviors of breast cancer cells through their interaction with the Wnt/β-catenin signaling pathway.

### 5.4 Promotion of apoptosis in breast cancer cells

Wolfiporia cocos (F.A. Wolf) Ryvarden and Gilb (Poria cocos), a well-known botanical drug, is highly regarded for its dual functionality, encompassing both its anti-cancer properties and its ability to enhance the immune system. The aforementioned approach has played a pivotal role in the management of breast cancer and the provision of quality care to patients. Pachymic acid has the ability to regulate the cell cycle of breast cancer cells in nude mice transplanted tumor model of MDA-MB-231 cells (ig, 700 mg/kg/day, 25 days). This modulation can be attributed to the downregulation of cyclinD1, cyclinE, cyclin-dependent kinase 2, and cyclin dependent kinase 4, along with the upregulation of p53 and p21 proteins. The series of events described ultimately leads to the activation of programmed cell death, known as apoptosis, in breast cancer cells (Jiang and Fan, 2020).

Matrine has been identified to inhibit the growth of breast cancer cells, specifically 4T1 and MCF-7 cells. It also prompts apoptosis in these cells in a dose- and time-responsive manner. This is achieved by suppressing the expression of vascular endothelial factors and down-regulating the Wnt/β-catenin signaling pathway (Xiao et al., 2018).

Saikosaponin D, which is a major metabolite of Bupleurum chinense DC. (Apiaceae; Bupleuri radix), has demonstrated the ability to inhibit the Wnt/β-catenin signaling pathway. This inhibition has been observed to induce apoptosis and decrease proliferation in HCC1937 cells, a specific subtype of TNBC (Wang et al., 2018a). Actinidia chinensis Planch. root extract metabolites have the ability to attenuate the phosphorylation of GSK-3β, leading to a subsequent decrease in the phosphorylation of β-catenin at specific residues. This phenomenon leads to the degradation of β-catenin, which subsequently inhibits the Wnt/β-catenin signaling pathway, thereby facilitating apoptosis in MDA-MB-231 and MDA-MB-453 breast cancer cells (MacDonald et al., 2009; Gan et al., 2021). Furthermore, some metabolites of TCM, such as triptolide (Shao et al., 2014), naringin (Li et al., 2013), and baicalein (Yu P. et al., 2023), have been discovered to induce apoptosis in breast cancer cells through the inhibition of β-catenin.

## 6 Potential of TCM formulas in breast cancer treatment

Traditional Chinese Medicine compounds are renowned for their multifaceted approach to treating malignant tumors. They target a plethora of sites, traverse various pathways, and manifest diverse therapeutic effects. One of the standout features of TCM is its minimal toxicity and side effects. Moreover, it bolsters the body’s immune defenses and presents a reduced risk of drug resistance, making it an increasingly attractive avenue for research in recent times. Empirical studies have underscored the efficacy of TCM in inhibiting cell proliferation and growth, curtailing cell migration and invasion, promoting cell apoptosis, and amplifying anti-inflammatory responses, primarily through the modulation of the Wnt/β-catenin signaling pathway (Table 3).

**TABLE 3:** TCM formulations targeting breast cancer treatment.

Formula	Form	Extraction method	Cell	Animal	Administration mode	Dose	Course of treatment	Contrast mode	Function	Mechanism	TCM theory	References
*XiaoPi* formul	Epimedium brevicornu Maxim. [Berberidaceae; Epimedii folium], Cistanche deserticola Ma [Orobanchaceae; Cistanches herba], Leonurus japonicus Houtt. [Lamiaceae; Leonuri herba], Salvia miltiorrhiza Bunge [Lamiaceae; Salviae miltiorrhizae radix et rhizoma], Curcuma aromatica Salisb. [Zingiberaceae; Curcumae radix], Curcuma phaeocaulis Valeton [Zingiberaceae; Curcumae rhizoma], Ligustrum lucidum W.T.Aiton [Oleaceae; Ligustri lucidi fructus], Reynoutria multiflora (Thunb.) Moldenke [Polygonaceae; Polygni multiflori radix], Ostreae Concha and Carapax trionycis	refluxing extraction method	4T1 cells	Mice	ig	1 g/kg/day	6 weeks	Normal saline (negative control)	Inhibiting value-added, and self-renewal activity	Chemokine (C-X-C) Ligand(CXCL)1↓,β-catenin↓	Clearing away heat and toxic material	Wang et al. (2017), Wang et al. (2020a), Zheng et al. (2020)
		refluxing extraction method and freeze-drying process	BT549 and 4T1 cells		ip	10, 20 mg/kg/day	—					
		Spin concentration, freeze drying	MDA-MB-231 and MCF-7 cells		ig	0.5 g/kg/dat		Normal mice (no-treatment control)				
*BaoYuan JieDu* decoction	Astragalus mongholicus Bunge [Fabaceae; Astragali radix], Panax ginseng C.A.Mey. [Araliaceae; Ginseng radix et rhizoma], Aconitum carmichaelii Debeaux [Ranunculaceae; Aconiti lateralis radix praeparata], *Lonicera japonica* Thunb. [Caprifoliaceae; Lonicerae japonicae flos], Angelica sinensis (Oliv.) Diels [Apiaceae; Angelicae sinensis radix] and Glycyrrhiza glabra L. [Fabaceae; Glycyrrhizae radix et rhizoma]	—	4T1 cells	Mice	ig	1 mL/10 g/day	2 weeks	Cisplatin (positive control) and normal saline (negative control)	Improve the ecological niche before transfer and extend the survival period	TGF- β/CCL9 signal pathway ↓,Wnt/β- catenin ↓	Strengthening body resistance and eliminating evil	Tian et al. (2020)
*XiHuang p*ill	Boswellia sacra Flück. [Burseraceae; Olibanum], Commiphora myrrha (T.Nees) Engl. [Burseraceae; myrrha], Moschus and Bovis Calculus	—	MCF-7, SKBr3 and MDA-MB-231 cells	Mice	ig	150 mg/kg/day	17 days	Normal saline (negative control)	Inhibition of proliferation, migration, and invasion	cAMP/PKA↑, AP-1↓, Wnt/β-catenin↓	Removing toxicity for eliminating carbuncles, Removing blood stasis, and knot	Hao et al. (2018), Su et al. (2018), Chen et al. (2023)
			4T1 cells	—	—	—	—	—				
				Mice	ig	0.2 mL, twice daily	14 days	Distilled water (negative control)				
*SanHuang Tang*	Rheum palmatum L. [Polygonaceae; Rhei radix et rhizoma], Scutellaria baicalensis Georgi [Lamiaceae; Astragali radix] and Coptis chinensis Franch. [Ranunculaceae; Coptidis rhizoma]	—	—	—	—	—	—	—	Reduce postoperative exudation of breast cancer	IL-2↑,IL-10↓,IL-β↓,TNF-α ↓	Clearing away heat and toxic material	Zhang et al. (2019a), Chen et al. (2021), Winardi et al. (2023)
			MCF-7 cells	Mice		6.4 g/kg/2 days	4 weeks	PBS (negative control)				
*RuYiPing*	Iphigenia indica (L.) A.Gray ex Kunth [Colchicaceae; Cremastrae pseudobulbus pleiones pseudobulbus], Curcuma phaeocaulis Valeton [Zingiberaceae; Curcumae rhizoma], Vespae Nidus, Coix lacryma-jobi var. ma-yuen (Rom.Caill.) Stapf [Poaceae; Coicis semen], Akebia quinata (Thunb. ex Houtt.) Decne. [Lardizabalaceae; Akebiae caulis] and Platycodon grandiflorus (Jacq.) A.DC. [Campanulaceae; Platycodonis radix]	—	4T1 cells	Mice	—	5.67, 22.68 g/kg/day	—	Clean water (negative control) and normal mice (no-treatment control)	Inhibition of breast cancer metastasis	IL-1β↓,IL-6↓,CXCL2↓,CXCL5↓	Strengthening body resistance and eliminating evil	Ye et al. (2019)
*QingDu* granule	Bupleurum chinense DC. [Apiaceae; Bupleuri radix], *Citrus reticulata* Blanco [Rutaceae; Citri reticulatae pericarpium], Curcuma aromatica Salisb. [Zingiberaceae; Curcumae radix], Styphnolobium japonicum (L.) Schott [Fabaceae; Sophorae flos], Paeonia × suffruticosa Andrews [Paeoniaceae; Moutan cortex], Arnebia euchroma (Royle ex Benth.) I.M.Johnst. [Boraginaceae; Arnebiae radix], Prunella vulgaris L. [Lamiaceae; Prunellae spica], Salvia miltiorrhiza Bunge [Lamiaceae; Salviae miltiorrhizae radix et rhizoma], Curcuma phaeocaulis Valeton [Zingiberaceae; Curcumae rhizoma], Astragalus mongholicus Bunge [Fabaceae; Astragali radix] and Glycyrrhiza glabra L. [Fabaceae; Glycyrrhizae radix et rhizoma]	80% ethanol extraction, rotary evaporation and freeze-dried	MCF-7 cells	Mice	ig	5.7, 18.0, 56.8 g/kg/day	21 days	Tamoxifen (positive control) and Carboxymethyl Cellulose (negative control)	Inhibiting tumor growth and anticancer vascular growth	Vascular Endothelial Growth Factor (VEGF)↓,VEGFR2↓,NFATc3↓,Wnt/β-catenin↓	Clearing away heat and toxic material	Zhao et al. (2018)
Compound *KuShen* injection	Sophora flavescens Aiton [Fabaceae; Sophorae fiavescentis radix] and Atractylodes macrocephala Koidz. [Asteraceae; Atractylodis macrocephalae rhizoma]	—	MDA-MB-231 cells	—	—	—	—	Tween 80 and HEPES (negative control)	Inhibiting proliferation and metastasis, Inducing apoptosis	TGF-β↓, GnRH↓, Adjusting VEGF and Wnt/β- catenin	Eliminating stagnation to stop pain	Xu et al. (2011), Nourmohammadi et al. (2019), Ye et al. (2022)
			MCF-7 and MDA-MB-231 cells					—				
			MCF-7 cells	Mice	ip	2 mL/kg/2 days	9 weeks	Cisplatin (positive control) and normal saline (negative control)				
*LiuWeiDiHuang* pill	Rehmannia glutinosa (Gaertn.) DC. [Orobanchaceae; Rehmanniae radix], Cornus officinalis Siebold & Zucc. [Cornaceae; Corni fructus], Dioscorea oppositifolia L. [Dioscoreaceae; Dioscoreae rhizoma], Paeonia × suffruticosa Andrews [Paeoniaceae; Moutan cortex], Poria and Alisma plantago-aquatica subsp. orientale (Sam.) Sam. [Alismataceae; Alismatis rhizoma]	—	MDA-MB-231 cells	Mice	po	2.3, 4.6 and 9.2 g/kg/day	—	Paclitaxel (positive control) and normal saline (negative control)	Suppress cell growth and metastasis to the lungs and liver	MAP3K1↑,KLF4↑,axin-2↑,TCF-1↓,β-catenin↓, Cyclin D1↓,VEGF↓	Strengthening body resistance and eliminating evil	Zheng et al. (2019b)
*ShuGanLiangXue* decoction	Paeonia × suffruticosa Andrews [Paeoniaceae; Moutan cortex], Schisandra chinensis (Turcz.) Baill. [Schisandraceae; Schisandrae chinensis fructus], Paeonia lactiflora Pall. [Paeoniaceae; Paeoniae radix alba], Vincetoxicum atratum (Bunge) C.Morren & Decne. [Apocynaceae; Cynanchi atrati radix et rhizoma], Bupleurum chinense DC. [Apiaceae; Bupleuri radix] and *Curcuma aromatica* Salisb. [Zingiberaceae; Curcumae radix]	Boiling and concentrating, and spray drying at low temperature and low pressure	MCF-7 and MDA-MB-231 cells	—	—	—	—	PBS (negative control)	Inhibiting the proliferation of breast cancer cells	Downstream target genes c-Myc and Bcl-2↓ of Wnt/β-catenin	remove blood stasis and collaterals	Zhou et al. (2018)
*TaoHongSiWu d*ecoction	Rehmannia glutinosa (Gaertn.) DC. [Orobanchaceae; Rehmanniae radix], Rehmannia glutinosa (Gaertn.) DC. [Orobanchaceae; Rehmanniae radix], Conioselinum anthriscoides “Chuanxiong” [Apiaceae; Chuanxiong rhizoma], Paeonia lactiflora Pall. [Paeoniaceae; Paeoniae radix alba], Prunus persica (L.) Batsch [Rosaceae; Persicae semen] and *Carthamus tinctorius* L. [Asteraceae; Carthami flos]	Decocting with ethanol, filtering, and concentrating the filtrate	MDA-MB-231 cells	—	—	—	—	—	Inducing apoptosis and inhibiting the proliferation of breast cancer cells	Regulate the RNA and protein expression of HRAS, MAPK1, AKT1, GRB2 and MAPK14	Blood circulation and blood stasis	Duan et al. (2020), Huang et al. (2021), Jiang et al. (2021), Gui et al. (2022)
			4T1 cells	Mice	ig	6.3, 12.6 and 25.2 g/kg, three times a day	18 days	Cisplatin (positive control), normal saline (negative control) and normal mice (no-treatment control)				
			MCF-7 and MDA-MB-231 cells			9.0 g/kg/day	7 days	Normal saline (negative control)				
*ZuoJinWan*	Coptis chinensis Franch. [Ranunculaceae; Coptidis rhizoma] and Tetradium ruticarpum (A.Juss.) T.G.Hartley [Rutaceae; Euodiae fructus]	—	4T1 cells	Mice	ip	1.8 g/kg/day	3 weeks	Cisplatin (positive control), normal saline (negative control) and normal mice (no-treatment control)	Inhibit the proliferation of breast cancer cells	Downstream target gene Cyclin D1↓ of Wnt/β-catenin	Grabbing fire open ruffian knot	Du et al. (2013), Wang et al. (2023b)

### 6.1 Inhibition of cell proliferation and growth

Many TCM prescriptions, as part of CAM, have been utilized in the treatment of breast cancer by clearing away heat and toxins, promoting blood circulation, and eliminating blood stasis, leading to positive outcomes. Research has found that the main mechanism of action of TCM formulas is to regulate the Wnt/β-catenin signaling pathway, which inhibits the growth and proliferation of breast cancer cells. For example, the *XiaoPi* formula demonstrates a notable capacity to suppress the activity of tumor-associated macrophages when co-cultured with breast cancer cell lines MDA-MB-231 and 4T1. This suppression results in a marked decrease in the proliferation and self-renewal capabilities of these cancer cells. Moreover, the *XiaoPi* formula can counteract the secretion of CXCL1 and β-catenin, further diminishing the self-renewal and chemotherapy resistance of the cancer cells (Zheng et al., 2020). *TaoHongSiWu* decoction’s serum appears to modulate the Wnt signaling pathway by regulating the RNA and protein expression of pivotal targets such as HRAS, MAPK1, AKT1, GRB2, and MAPK14. This interaction with the β-catenin signaling pathway curtails cell proliferation in breast cancer cell lines MCF-7 and MDA-MB-231, with the degree of inhibition being both time and concentration-dependent (Huang et al., 2021).

### 6.2 Suppression of cellular migration and invasion

Most breast cancer is invasive and often metastasizes to lymph nodes and then to distant organs, including the bone, lungs, liver, and brain. At the same time, tumor metastasis is also the primary cause of death among breast cancer patients. Many studies have found that TCM prescriptions can regulate the Wnt/β-catenin signaling pathway, improve the microenvironment before breast cancer metastasis, and inhibit the metastasis ability of breast cancer cells. *BaoYuanJieDu* decoction offers another avenue of therapeutic potential. It acts by inhibiting the TGF-β/CCL9 signaling pathway, subsequently disrupting the Wnt/β-catenin signaling. This disruption is particularly significant as it hinders the recruitment of myelogenous suppressor cells to the lungs, a common site for breast cancer metastasis. By improving the pre-metastatic microenvironment, *BaoYuanJieDu* decoction not only hinders metastasis but also prolongs the survival of mice bearing 4T1 tumors (ig, 1 mL/10 g/day, 2 weeks) (Tian et al., 2020).

In a study conducted by Zhang et al. (2019), it was observed that the administration of the *LiuWeiDiHuang* pill resulted in the upregulation of axin-2, while simultaneously downregulating TCF-1, β-catenin, cyclin D1, and VEGF in TNBC bearing-mice (po, 2.3 g/kg/day). This modulation hinders the activation of the β-catenin/TCF-1 pathway, consequently diminishing the metastatic capacity of TNBC cells, specifically towards organs such as the lungs and liver (Zheng L. X. et al., 2019). Furthermore, there is clinical evidence indicating that women diagnosed with type 2 diabetes have a higher susceptibility to developing breast cancer compared to women without diabetes. The study revealed a correlation between elevated blood sugar levels and the development of breast cancer in breast cells (Michels et al., 2003). Additionally, it was found that the administration of the *LiuWeiDiHuang* pill, a TCM, can mitigate the risk of breast cancer by effectively managing diabetes (Wu C. T. et al., 2018).

*RuYiPing* is a frequently employed compound medication for the clinical management of metastatic breast cancer. Ye et al. (2019) discovered that the combination of *RuYiPing* and Platycodon grandiflorus (Jacq.) A.DC. (Campanulaceae; Platycodonis radix) has the potential to decrease the expression of IL-1β, IL-6, CXCL2, and CXCL5, which in turn helps to preserve vascular integrity in 4T1 tumor-bearing mice (5.67 and 22.68 g/kg/day, 14 days). This mechanism ultimately inhibits the pre-metastatic microenvironment in the lungs for breast cancer (Ye et al., 2019).

### 6.3 Further insights

Traditional Chinese medicine prescriptions follow the principle of “Jun Chen Zuo Shi” and typically consist of a variety of TCM components, each capable of exerting diverse therapeutic effects on specific targets. Research has found that TCM prescriptions can not only have therapeutic effects on breast cancer but also can treat some postoperative complications of the disease. For example, Winardi et al. (2023) discovered that the administration of *SanHuangXieXin* Decoction resulted in a notable decrease in patient mortality. Furthermore, patients with breast cancer who received the compound medicine exhibited lower mortality rates compared to those who solely received single ingredient treatments (Winardi et al., 2023). Simultaneously, *San Huang Tang* has demonstrated its efficacy in reducing inflammatory markers, including TNF-α, IL-6, IL-8, and c-reactive protein. By modulating these markers, it indirectly influences the Wnt/β-catenin signaling pathway. This modulation has been linked to a notable decrease in postoperative exudation and an enhancement in post-surgical inflammation management for breast cancer patients (Chen et al., 2021).

Another promising combination involves *TaoHongSiWu* Decoction paired with neoadjuvant chemotherapy, specifically the CAF/CEF or TAG/TEC regimens. This combination has been observed to effectively curb both tumor lymphangiogenesis and angiogenesis in breast cancer patients. The underlying mechanism is believed to involve the suppression of VEGF-C and VEGF-A expression, along with alterations in lymphatic vessel density and microvessel density. Moreover, the combined influence of *TaoHongSiWu* Decoction and chemotherapy drugs leads to a downregulation in the Wnt/β-catenin pathway, specifically targeting the downstream gene bcl-2. This results in an upregulation of the bax protein, a decrease in the bcl-2/bax ratio, and the induction of tumor cell apoptosis. At the same time, *TaoHongSiWu* Decoction is effective in treating upper limb swelling after breast cancer surgery, and the cure rate can reach 87.9%, showcasing its potential in treating invasive breast cancer (Jiang et al., 2021).

## 7 Biomarkers for breast cancer treatment using TCM based on key proteins in the Wnt pathway

Traditional Chinese Medicine has shown significant potential in breast cancer treatment, yet only a handful of TCM formulations are widely used in clinical practice. To gain broader acceptance and application for TCM in breast cancer therapy, rigorous scientific research and validation are essential.

A key challenge in TCM is the variability of ingredients in herbal formulations, making standardization and quality control paramount. Unlike the quality control methods for foreign herbal medicines, which primarily focus on chemical qualitative identification and indicator components, TCM’s complexity demands a more nuanced approach. TCM’s efficacy cannot be fully captured by chemical analysis alone due to its multi-component, multi-target nature, and the intricacies of its effects and compatibility (Wu X. et al., 2018). Research has found that biomarkers derived from metabolomics offer advantages in terms of completeness, systematicity, and quantification. They also exhibit strong discriminative power in assessing the quality of TCM. The quality evaluation of TCM can be directly and simultaneously examined from both chemical and biological aspects. The quality evaluation of TCM reflects the effectiveness and safety of this practice. Moreover, biomarkers can simplify the evaluation of the quality of TCM by offering direct indicators of biological impact (Wu X. et al., 2018; Gao et al., 2020). Thus, biomarkers have emerged as vital indicators of biological activity, offering a novel approach to assessing the safety and efficacy of TCM (Giridhar and Liu, 2019; Wu and Chu, 2021). These biomarkers, with their high specificity and sensitivity, are instrumental not only in evaluating TCM quality but also in monitoring its quality fluctuations. For example, research has shown that endogenous biomarkers such as LysoPC (22:5), valine, and shikimic acid can be utilized to assess the toxic components of Aconitum carmichaelii Debeaux (Ranunculaceae; Aconiti radix) (Zhou et al., 2016). Schisandrol A, schisandrin A, schisandrin C, and gomisin N are considered biomarkers for evaluating the quality standard of Schisandra chinensis (Turcz.) Baill. (Schisandraceae; Schisandrae chinensis fructus) (Zhang Y. et al., 2019). In the Chinese Pharmacopoeia, evodiamine is designated as a biomarker for evaluating the quality standards of Tetradium ruticarpum (A.Juss.) T.G.Hartley (Rutaceae; Evodiae fructus) and Chinese medicines containing this plant (Yang W. et al., 2017). The effectiveness of the *Lianhua Qingwen* Capsule can be assessed and managed using potential biomarkers such as L-ornithine, prostaglandin F_2α_, and arachidonic acid (Gao et al., 2020).

In addition, biomarkers can also serve as quality standards for evaluating the effectiveness of TCM in treating various diseases. For example, LysoPC (16:0), leucine, glutamine, 5-hydroxytryptamine, and other potential biomarkers are used to assess the quality of *Wuda* granule as a therapeutic agent for promoting recovery after surgical resection of colorectal cancer (Wang T. et al., 2020). Potential biomarkers such as glucose, lactic acid, and triglycerides can be used to evaluate the effectiveness of Salvia miltiorrhiza Bunge (Lamiaceae; Salviae miltiorrhizae radix et rhizoma) in treating colorectal cancer. Meanwhile, biomarkers such as glutathione, glyoxylate, and inosine are effective tools for assessing the potential anti-fatigue effects of Salvia miltiorrhiza Bunge (Lamiaceae; Salviae miltiorrhizae radix et rhizoma) (Wang Y. et al., 2021). Biomarkers such as D-galactose, inositol, and glycolol can be used to assess the effectiveness of Aconitum carmichaelii Debeaux (Ranunculaceae; Aconiti radix) in combination with Ampelopsis japonica (Thunb.) Makino (Vitaceae; Ampelopsis radix) for treating rheumatoid arthritis (Jin H. et al., 2019). Glycocholic acid, taurocholic acid, and indole acetate are considered potential biomarkers for evaluating the therapeutic effect of *DaHuangXiaoShi* decoction on cholestasis (Zhu and Feng, 2019). Biomarkers such as 2-ketobutyric acid, 3-hexenedioic acid, and argininic acid can be utilized as indicators to assess the therapeutic efficacy of Gout Party in the management of acute gouty arthritis (Wang et al., 2019).

For breast cancer treatment, TCM increasingly relies on biomarkers for standardization. Tumor biomarkers, produced by interactions between tumor tissues or the host and the tumor, are key in indicating tumor presence and progression (von Voithenberg et al., 2019). An ideal tumor biomarker should satisfy three key criteria: analytical validity, clinical validity, and clinical utility. Analytical validity necessitates that biomarkers exhibit accuracy, sensitiv patity, specificity, and stability. Clinical validity involves the biomarker’s capability to detect the disease’s status and project outcomes. Clinical utility implies that using the biomarker should improve patient outcomes compared to scenarios where it is not used (Merker et al., 2018).

Potential biomarkers of breast cancer, such as L-Arginine, arachidonic (Mao C. et al., 2022), urea (Nam et al., 2009), and palmitic acid (Tan et al., 2020), have been proven to be highly correlated with the Wnt/β-catenin signalinghway. The key protein components of this pathway, including β-catenin (Abu El Abbass et al., 2020), APC (Wang X. C. et al., 2021), c-Myc (Liu et al., 2021; Gao et al., 2023), Bcl-2 (Kolecková et al., 2017), and cyclin D1 (Liu N. Q. et al., 2022), are related to the occurrence, progression, and metastasis of breast cancer. They meet the criteria of tumor biomarkers and are expected to become potential prognostic and predictive biomarkers of breast cancer. Utilizing biomarkers to assess TCM quality is a critical step towards enhancing its existing quality control standards and achieving standardization. Therefore, the relationship between TCM and the Wnt/β-catenin signaling pathway is integral to developing a standardized and robust quality evaluation system for TCM, particularly in the context of breast cancer treatment.

## 8 Conclusion and outlook

Recently, there has been a notable surge in the integration of TCM into the comprehensive care of cancer patients, particularly in the postoperative and adjuvant stages of those undergoing radiotherapy and chemotherapy. Beyond its conventional uses, TCM extends its therapeutic reach by addressing the adverse effects that often follow breast cancer surgery, radiotherapy, and chemotherapy, utilizing methods such as dietary therapy and acupoint application. This integrative approach has yielded promising therapeutic outcomes. Contrasting with the often harsh nature of conventional radiotherapy and chemotherapy, TCM is characterized by its gentler approach, typically resulting in fewer side effects and reduced toxicity. This makes it an appealing option for enhancing patient comfort, alleviating pain, and potentially improving life expectancy. An abundance of research highlights the diverse mechanisms by which TCM can effectively combat tumors, offering a wider range of therapeutic options for managing breast cancer. This expanded repertoire of treatments underscores the growing significance of TCM in the holistic care of breast cancer patients.

This review delves into the influence of Chinese medicine monomers and compounds on various aspects of breast cancer cell dynamics, from cell cycle regulation and proliferation to migration, invasion, and apoptosis, all through the lens of the Wnt/β-catenin signaling pathway. The insights gleaned from this exploration aim to lay the groundwork for the future design of innovative drugs tailored to specific breast cancer subtypes or particular phases of the Wnt/β-catenin signaling cascade.

## Abbreviations

AcRoots, Actinidia chinensis Planch. Root; BCSCs, Breast Cancer Stem Cells; CAM, Complementary and Alternative Medicine; CXCL, Chemokine (C-X-C) Ligand; EMT, Epithelial-Mesenchymal Transition; ER, Estrogen Receptor; GSK-3β, Glycogen Synthase Kinase-3β; HER-2, Human Epidermal Receptor 2; LRP6, Low Density Lipoprotein Receptor-Related Protein 6; MaSCs, Mammary Stem Cells; PR, Progesterone Receptor; TCM, Traditional Chinese Medicine; TGF-β, Transforming Growth Factor-β; TNBC, Triple-Negative Breast Cancer; TNF-α, Tumor Necrosis Factor-alpha; VEGF, Vascular Endothelial Growth Factor.
